# Influence of Photoperiod on Membrane Fouling Reported in a Membrane Photobioreactor for Reclamation of Anaerobic Effluent

**DOI:** 10.1021/acsestwater.5c00685

**Published:** 2026-02-16

**Authors:** Elvira Ferrera, Ignacio Ruigómez, Cristina González-Martín, Cristian Mejías, Luisa Vera

**Affiliations:** † Departamento de Ingeniería Química y Tecnología Farmacéutica, Facultad de Ciencias-Sección de Química, 16749 Universidad de La Laguna, Av. Astrof. Fco. Sanchez s/n, Apdo. 456, 38200 La Laguna, Tenerife, Spain; ‡ Departamento de Obstetricia y Ginecología, Pediatría, Medicina Preventiva y Salud Pública, Toxicología, Medicina Legal y Forense y Parasitología. Instituto Universitario de Enfermedades Tropicales y Salud Pública de Canarias. Universidad de La Laguna, 38200 La Laguna, Tenerife, Spain

**Keywords:** microalgae−bacteria consortia, membrane fouling, upflow anaerobic sludge blanket, domestic wastewater, photosynthetic activity

## Abstract

The combination of anaerobic and membrane photobioreactor (MPBR) technologies has generated interest as a sustainable strategy for domestic wastewater treatment. The former allows lower sludge production than aerobic technologies and energy recovery through biogas production, while MPBRs act as an advanced secondary effluent treatment. This study shows the effects of the light photoperiod on the suspension characteristics and overall performance of bench-scale MPBRs applied to an advanced secondary effluent treatment of a pilot upflow anaerobic sludge blanket (UASB). The results of the experimental runs showed the key role played by light incidence on the organisms developed and consequently on wastewater recovery performance and membrane fouling. The two light/dark photoperiods tested (9/15 h and 12/12 h) resulted in similar values for regeneration performance and membrane fouling. However, the longer photoperiod promoted a more sustainable process of nitrogen conversion associated with photosynthetic activity. In addition, longer daylight hours contributed to membrane performance characterized by more easily removable fouling using physical cleaning methods and with less residual character. Consequently, the tested technology can further improve performance in terms of long-term membrane operation and contributes to the design of an overall sustainable process.

## Graphical abstract

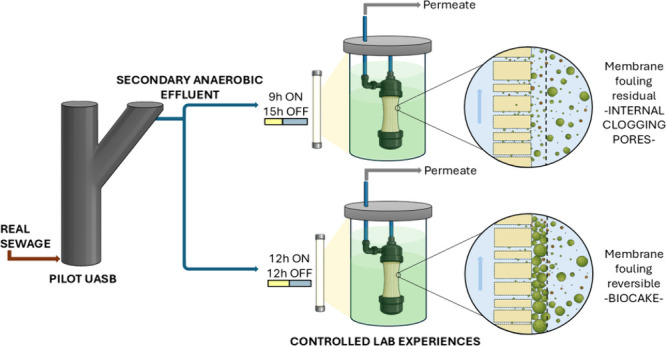

## 1 Introduction

Anaerobic treatment of domestic wastewater has gained great importance in recent years due to the increasing energy prices and the new circular economy paradigm that also involves wastewater treatment processes. Some advantages of anaerobic technologies are their capacity for partial organic matter degradation without oxygen. Anaerobic treatments also enable the recovery and energetic valorization of biogas. Additionally, there is less sludge production than conventional aerobic processes.^1,2^ One of the anaerobic technologies which is receiving renewed attention is the upflow anaerobic sludge blanket (UASB).^3^ Nevertheless, limited ammonium removal and phosphorus recovery efficiency impede its use in some cases, requiring post-treatment of anaerobic effluents to achieve the legal discharge requirements.^4^ In fact, several previous studies have focused on integration of UASB reactors with aerobic post-treatment, such as membrane bioreactors (AeMBRs).^5–8^ These systems offer a promising opportunity for ammonium removal through partial nitritation/nitrification processes, with reported removal efficiencies of 98% for organic matter and from 39% to 86% for total nitrogen, depending on the operating conditions employed.^8,9^

An alternative technological option could be the combination of UASBs with photobioreactor technology (PBR) due to their potential for advanced biological remediation of wastewater.^10,11^ This is interesting because it could contribute to reducing the risk of eutrophication, provided that the biomass efficiently accumulates nutrients and is adequately separated from the treated effluent, thereby preventing nutrient discharge and subsequent negative impacts on receiving aquatic ecosystems.^4^ In addition, water reuse for purposes such as crops, gardens or golf courses irrigation improves if compounds released can be controlled from regenerated wastewater.^12,13^ Many authors have demonstrated the successful application of photobioreactors for microalgae cultivation in wastewater to remove pollutants,^10,14,15,17^ but most of the referenced studies were developed with monocultures to compare their capabilities for nutrient removal or specific compound generation.^18^ However, maintaining a microalgae monoculture under realistic conditions in wastewater reclamation facilities is difficult, and several studies have reported both competitive and cooperative interactions between microalgae and bacteria.^19^ In this sense, the fact that microalgae are photoautotrophic implies that the involvement of bacteria is necessary to achieve substantial removal of organic carbon from wastewater.

The most common large-scale culture systems of algae are open ponds and photobioreactors. The latter often show improved photosynthetic efficiency and a lower footprint but also have some disadvantages, such as poor sedimentation, biomass washout, and harvest limitations. To address the challenges of PBR operation, a new concept has recently emerged: the membrane photobioreactor (MPBR), which integrates PBRs with membrane filtration processes, usually ultrafiltration membranes.^20^ Chen et al.^21^ demonstrated the potential of MPBR equipped with a microfiltration membrane for nutrient removal from an anaerobic digestion effluent. This study, conducted under controlled conditions at lab scale, showed the capacity of an MPBR to eliminate the inhibitory effects of suspended solid and microorganisms and maintain high microalgae concentrations as well as achieving high ammonia and phosphate removal. It has been reported that MPBRs can produce highly concentrated biomass, 3.5 times higher than PBRs, and achieve significant nutrient removal mainly due to the independent control of HRT and SRT.^22,23^ However, most reported experimental studies on MPBR have been developed on a laboratory scale with artificial wastewater and microalgae monocultures.^24,25^ On the other hand, although their integration as a post-treatment for nutrient recovery from effluents generated by aerobic processes has been explored, their application following anaerobic systems has scarcely been investigated, and always in combination with AnMBR systems.^26–30^ The latter provides secondary effluents free of suspended solids with high concentrations of N and P, although they are also associated with high operational and maintenance costs. In this context, the use of UASB reactors in subtropical regions such as the Canary Islands is particularly relevant, since temperatures remain moderate throughout the year and daylight hours do not decrease significantly in winter. This scenario represents an opportunity to evaluate the combination of low-cost anaerobic treatments with advanced microalgae-based systems, which could overcome the drawbacks associated with AnMBRs.^31^ However, the presence of particulate matter in the MPBR feed constitutes a significant challenge, as it can cause severe membrane fouling and reduce light penetration in the suspension that leads to lower photosynthetic activity of phototrophic species.^3,32^

In summary, despite their advantages, the main limitation of MPBRs remains membrane fouling, and their performance depends on the proper foulants mitigation and control to ensure efficient filtration.^33,34^

Therefore, maintaining the membrane properties during the separation process is a key challenge for MPBR implementation.^23,35^ Indeed, the sustainability of MPBRs requires the design of adequate strategies for effective membrane fouling control and mitigation. According to the origin of the clogging species, membrane fouling can be biological, organic or inorganic.^36^ The growth and evolution of microalgae and their corresponding metabolic processes are also relevant to understanding MPBR performance. Abiotic factors include wastewater characteristics, as well as operational (hydraulic retention time (HRT), biological solids retention time (SRT), biomass concentration) and environmental factors (temperature, CO_2_, available light).

Currently, there is still a lack of knowledge in the literature regarding the application of MPBRs operated with microalgae–bacteria consortia as an advanced treatment of anaerobic secondary effluents characterized by high ammonium concentrations.

The scientific contribution of this research lies in evaluating the performance of a laboratory scale MPBR fed with a real effluent from a pilot-scale UASB reactor operated in continuous mode. The study investigates the influence of distinct photoperiods, representing both favorable and unfavorable irradiations on biomass dynamics. From the results, it would be possible to estimate the availability of photoautotrophic microorganisms’ culture in turbid waters and under poor light irradiation. In addition, the level of reclamation achieved and membrane fouling behavior reported from each assessed photoperiod were analyzed. By addressing this gap in knowledge, the work offers new insights into the feasibility and limitations of MPBR technology for the advanced treatment of anaerobic effluents.

## 2 Material and Methods

### 2.1 Feedwater Characteristics

The experimental laboratory units were fed with anaerobic effluent from a UASB pilot unit located in the Tenerife Northeast Municipal Wastewater Treatment Plant (WWTP) (Canary Islands, Spain). The UASB was operated under psychrophilic conditions (average temperature of 18.5 ± 1.2 °C), feeding primary effluent from the WWTP consisting of desanding, degreasing and fine screening as described by Ferrera et al.^37^ Table 1 shows the average values of the main parameters of the UASB effluent, which was continuously fed to the bench-scale units during the experimental runs. The effluent composition, particularly the relatively high COD concentration, reflects the representative performance of this specific UASB reactor, which is aligned with or exceeds values typically reported for municipal UASB systems for sewage treatment.^31,38^

**Table 1:** Main Characteristics of Feedwater

		mean ± SD	
parameter	units	R1 and R2	R3
pH	–	7.9 ± 0.3	7.8 ± 0.3
turbidity	NTU	52.9 ± 25.1	74.9 ± 27.6
CE	μS/cm	1739.4 ± 155.2	1697.3 ± 270.1
TSS	mg/L	47.6 ± 13.3	30.4 ± 14.1
VSS	mg/L	41.2 ± 13.3	30.1 ± 13.8
HCO_3_^–^	mg/L	150.4 ± 13.7	149.4 ± 23.3
COD	mg/L	187.7 ± 44.5	143.2 ± 41.8
COD_s_	mg/L	114.9 ± 18.5	89.8 ± 23.2
DOC	mg/L	41.5 ± 6.2	36.6 ± 9.1
N-NH_4_^+^	mg/L	65.5 ± 5.8	64.9 ± 3.8
TP	mg/L	12.3 ± 1.6	10.9 ± 1.7

### 2.2 Selection of Photoperiods

The hours of light/darkness for the location of the Northeast WWTP in Tenerife (Canary Islands, Spain) were analyzed during a typical year between the period 2005–2023. Data on solar radiation were obtained from the Photovoltaic Geographical Information System (PVGIS) web application, which calculates solar radiation to a precision of 1 km^2^. Based on these data, the daylight hours of each day and month for a typical year were considered to obtain the average daily light/darkness ratio for each month (Table 2). This enabled us to define the two photoperiods applied to the bench-scale runs: the 9/15 photoperiod was defined as typical for the November–April period, and the photoperiod 12/12 was representative for May–October, as an example of the spring-autumn lighting, commonly used in the literature.^39,40^ Therefore, three experimental trials were conducted in this study: R1, R2, and R3. Trial R1 was carried out with a photoperiod of 9 h of light and 15 h of darkness, while trials R2 and R3 were conducted with a 12:12 h light/dark cycle. The difference between R2 and R3 lies in the mixing strategy: while R2 was carried out with aeration at the bottom of the tank, R3 was carried out without aeration, and instead, mechanical agitation was used. Hence, this work should be considered as a preliminary step in the effort to scale up the combination of UASB and MPBR technologies to the pilot level for future evaluation under more complex environmental scenarios.

**Table 2:** Average Monthly Photoperiods Obtained from the Northeast WWTP

months	photoperiod (light/dark (h))	average photoperiod (light/dark (h))	runs
November	7/17	9/15	R1
December	9/15		
January	9/15		
February	7.5/16.5		
March	9.5/14.5		
April	9.5/14.5		
May	12.5/11.5	12/12	R2, R3
June	13/11		
July	13/11		
August	13/11		
September	10/14		
October	11/13		

### 2.3 Membrane Photobioreactor Unit

The membrane photobioreactor had a working volume of 2.8 L and was equipped with an Immersed ZeeWeed ZW-1 ultrafiltration module (SUEZ Water Technologies and Solutions, Canada). This lab module is made up of a bundle of hollow PVDF fibers with an external diameter of 1.9 mm, a mean pore diameter of 0.4 μm, and a filter surface of 0.047 m^2^ (Figure 1). The permeate was continuously extracted at constant flux with a double-direction microgear magnetic pump, for filtration and backwashing processes. During the experimental period, a constant permeate flux of 10 L/h·m^2^ was maintained during 450 s, and a backwash flux of 30 L/h·m^2^ during 30 s was applied between filtration cycles. Depending on the configuration, bottom aeration supplied by a compressor (Secoh–Shanghai Mec, Japan–China) or either a magnetic stirring plate model RCT Basic (IKA, Germany) operated continuously at 150 rpm at the base of the unit was used to ensure homogenization and prevent biomass settling, since one of the main aims of the present work was to investigate the influence of biomass dynamics on membrane fouling. In addition, an Easy-Load Masterflex (Cole-Parmer, USA) peristaltic pump was used to keep the HRT constant, extracting part of the permeate and recirculating the rest to the photobioreactor. All sensors and operational parameters were monitored and controlled by using DAQ Factory software (AzeoTech, Inc.). The transmembrane pressure (TMP) was measured by using a pressure transducer (BD SENSORS, Germany) and recorded in the software every 10 s, which enabled accurate monitoring and analysis of membrane fouling. During long-term tests, continuous TMP recording allowed the identification of two parameters widely used to characterize fouling in filtration modules (Figure 2): the initial TMP (TMP_i_) and the final TMP (TMP_f_).^41,42^ TMP_i_ corresponds to the transmembrane pressure recorded at the beginning of the filtration cycles and is associated with internal residual fouling. This type of fouling is mainly caused by colloidal or soluble species, which may lead to phenomena such as gel layer formation, adsorption, or pore blocking, difficult to remove by physical cleanings. TMP_f_, on the other hand, represents the total fouling of the membrane at the end of each filtration cycle, i.e., just before physical cleaning. This value reflects the cumulative effect of both reversible and irreversible fouling.

**Figure 1:**
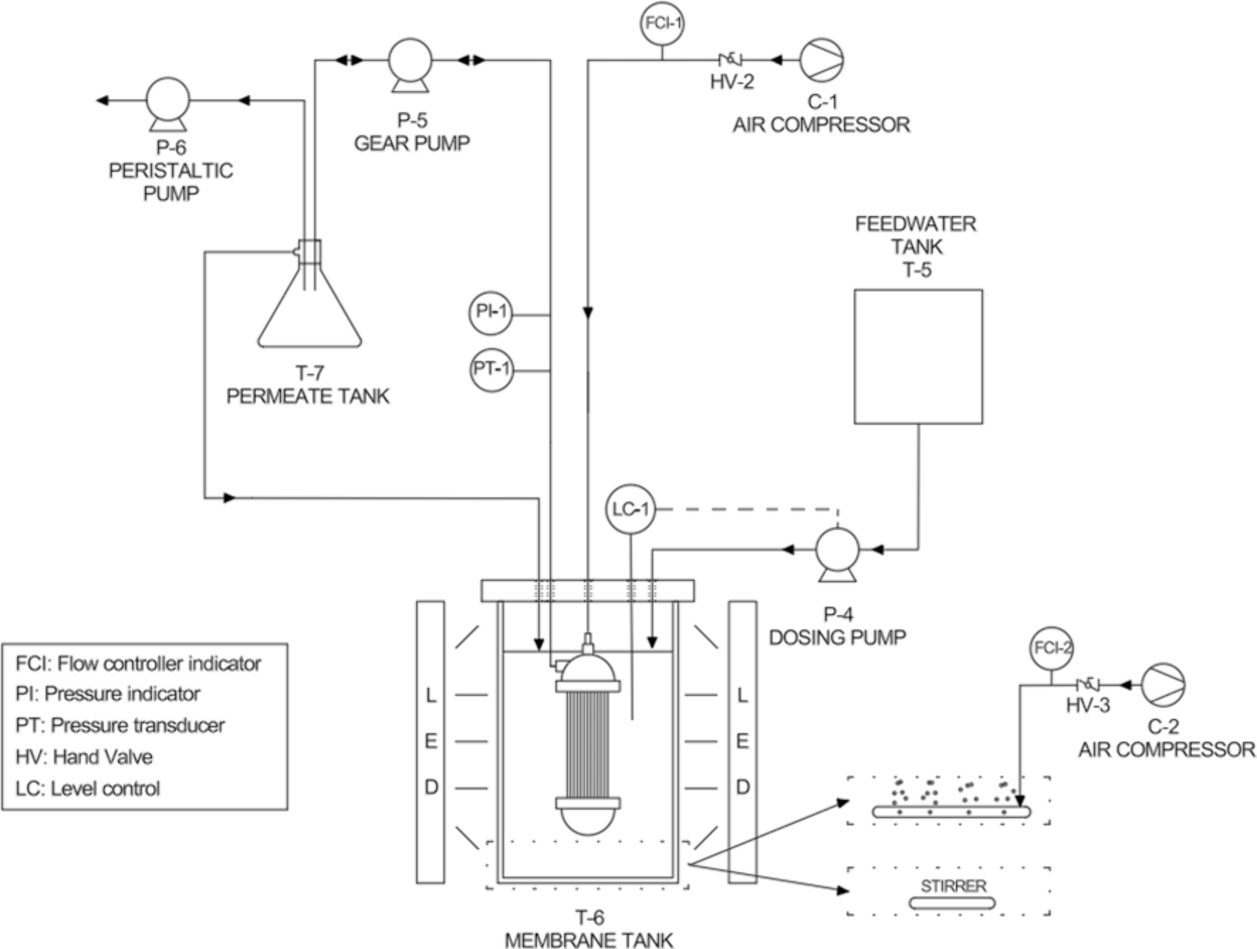
Schematic of the MPBR laboratory unit with pneumatic agitation by aeration in the bottom of the membrane tank (R1 and R2 experimental runs) or by magnetic stirring (R3 experimental run) for mixing the suspension.

**Figure 2:**
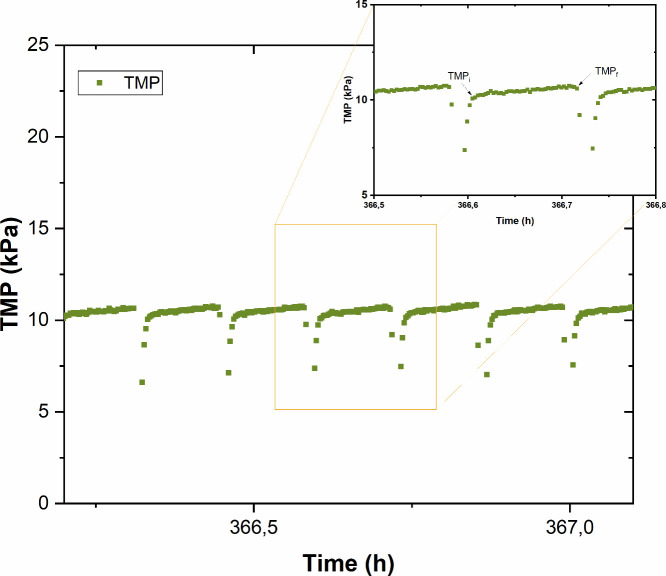
TMP profiles under consecutive filtration/backwashing cycles, where TMP_i_ and TMP_f_ are defined.

Additionally, an EasyLed Universal model lamp (Aquatlantis, Portugal) was used to promote microalgae growth under the preselected light/dark photoperiods of 9/15 and 12/12. This lamp is supported inside a plastic chamber covered by a reflective material to maximize light dispersion toward the MPBR, emitting light intensity at 215 μmol/m^2^·s. The membrane photobioreactor temperature was maintained at 18.5 ± 1.2 °C.

Both experiments were carried out at HRT and SRT of 3.5 and 9 days, respectively, which are into the typical ranges of previous studies.^43–45^ In addition, it should be highlighted that the MPBR was operated without an inoculum, so microalgae were grown until reaching a concentration of approximately 650 mg/L VSS at the 12/12 photoperiod. This concentration is within the typical range of microalgae concentrations in MPBRs for sewage treatment and does not limit light penetration according to previous studies.^24,46^ After the acclimation period, the biomass was periodically and manually purged to keep the SRT constant during the experimental tests, which were conducted under the photoperiods described in section 2.2.

### 2.4 Analytical Methods

Chemical oxygen demand (COD), total suspended solids (TSS), volatile suspended solids (VSS), pH and electrical conductivity (EC) were determined according to standard methods.^47^ Ion chromatography was used to determine the concentrations of dissolved nitrogen species: ammonium (NH_4_^+^), nitrites (NO_2_^–^) and nitrates (NO_3_^–^) using the Methrom 882 compact ion chromatography instrument (Methrom, Switzerland). The soluble COD was measured from filtered samples using 1.2 μm, and the difference in the COD between the filtered supernatant of the suspension and the permeate was used to determine the concentration of biopolymer clusters (BPC). Total phosphorus was measured with the LCK350 kit (Hach Lange GmbH, Willstätterstraße, Germany), and the dissolved organic carbon (DOC) was obtained from previously filtered samples at 1.2 μm with a multi N/C 3100 equipment (Analytik Jena GmbH), which consists of a catalytic oxidation at 850 °C, VITA Flow Management System. The gases resulting from oxidation are recorded by using a Focus Radiation NDIR – Detector. Dissolved oxygen (DO) was measured using an oximeter (Hach Lange LDO, USA). In addition, the particle size distribution of feedwater and mixed-liquor was determined with a Malvern Mastersizer 2000 using the small-volume entry-level wet dispersion unit Hydro 2000 SM (Malvern Instruments Ltd., U.K.). Furthermore, biomass characterization was conducted by thermogravimetric analysis using a Discovery SDT 650 instrument (TA Instruments, USA). Samples of 4–6 mg were analyzed under a nitrogen flow of 0.05 L/min and a heating rate of 9.85 °C/min. The TG and DTG curves were evaluated over the temperature range of 200–500 °C, applying a symmetric Gaussian curve model for each pseudocomponent, following the methodology of Ferreira et al.^48^ Biomass productivity (BP) was estimated from the mixed liquor volatile suspended solids (MLVSS) and the solid retention time (SRT), described by^44^

1 $$\mathrm{BP}=\frac{\mathrm{MLVSS}}{\mathrm{SRT}}$$

Nitrogen and phosphorus recovery rates (NRR and PRR) were calculated from influent and effluent concentrations, reactor feed flow rate, and reactor volume, described by González-Camejo et al.:^44^

2 $$\mathrm{NRR}=\frac{Q\cdot \left(N_{i}-N_{e}\right)}{V}$$

3 $$\mathrm{PRR}=\frac{Q\cdot \left(P_{i}-P_{e}\right)}{V}$$

### 2.5 Membrane Cleaning Protocol and Fouling Characterization

After the long-term experiments, a cleaning protocol was carried out to study the fouling layers generated inside and on the membrane. This protocol was performed following the previous one defined by Ruigómez et al.:^49^ (1) rinsing with tap water, using 4 L; (2) continuous backwashing at a flux rate of 30 L/h·m^2^ with 1.4 L of distilled water; (3) chemical cleaning with sodium hypochlorite solution (500 mg/L) for 24 h; (4) chemical cleaning with a citric acid solution (6 g/L) for 2 h; (5) continuous backwashing with sodium hypochlorite solution (500 mg/L); (6) chemical cleaning with sodium hypochlorite solution (1 g/L) for 12 h. After each step, a water filtration test was performed to determine the remaining transmembrane pressure (TMP), from which the remaining membrane resistance was calculated.

### 2.6 Microbiological Identification Method

The microbiological culture was studied using optic microscopy following the protocol described in ref 50 with some modifications. Briefly, the sample from the reactor was gently shaken for homogenization, and an aliquot of 100 μL was deposited on a microscope slide and the coverslip was placed on it, pressing gently. Three replicates were analyzed for each sample using a Leica DM750 microscope with different objectives (4×, 10×, 40×, 100×). The species and groups of microorganisms (ciliates, flagellates, rotifers, etc.) were identified using available manuals.^51,52^ The photosynthetic microorganisms of interest (cyanobacteria, microalgae, and diatoms) were counted, with a minimum of at least 10 counts per preparation, and the mean was calculated considering each replicate and objective. Results are expressed as microorganisms per milliliter. During the experimental runs, suspension samples were taken to identify the evolution of photosynthetic microorganisms from the beginning of each run.

In addition, respirometric tests were carried out at the end of each run to assess the photosynthetic activity or the dark respiration of microalgae and bacteria cultures following the standardized procedure by Rossi et al.^53^ This protocol is designed to distinguish among microalgal photosynthesis, heterotrophic respiration, and nitrifying activity. In S-I (30 min, light with ammonium and nitrite addition), the overall metabolic activity is stimulated, allowing simultaneous microalgal photosynthesis and bacterial (nitrifying and heterotrophic) respiration. S-II (10 min, dark, without inhibitors) measures total respiratory oxygen consumption from all microbial groups. In S-III (10 min, dark with allylthiourea and chlorate), the nitrifying activity is inhibited to isolate the respiration of microalgae and heterotrophs. S-IV (15 min, light under inhibition) quantifies net photosynthetic oxygen production from microalgae without interference from the nitrifiers. Finally, S-V (10 min, dark with inhibitors) assesses residual respiration attributable solely to microalgae and heterotrophs. Dissolved oxygen data from each phase are used to calculate the oxygen production and consumption rates, enabling the differentiation and quantification of the main functional contributions within the consortium.

## 3 Results and Discussion

### 3.1 Effects of the Light Photoperiod on Biomass

#### 3.1.1 Biomass Physicochemical Characterization

Table 3 summarizes the main characteristics of the suspensions developed in the MPBR during the two experimental runs. In both suspensions, the pH remained stable at values lightly above neutrality (7.8 ± 0.3 and 7.9 ± 0.3 for R1 and R2, respectively), thus preventing ammonium loss from the suspension due to stripping phenomenon. Additionally, during the acclimation phase (12/12 light/dark photoperiod without biomass purge), both experimental series exhibited a similar upward trend in biomass productivity (BP), reaching values of approximately 85 mg VSS/L·d after 600 h of experimentation (Figure 3). At that point, both units reached the target VSS concentration (650 mg/L), and the purge pump was activated, leading to a progressive decrease in BP. This change in trend was driven by the reduction in the SRT from infinity to 9 days in both experiments. The decrease in biomass productivity was much more pronounced in R1 than in R2 when the photoperiod was set to the experimental operating conditions (light/dark photoperiod of 9/15). It is known that the photoperiod and intensity to which an algal-bacterial consortium is exposed can significantly affect the ratio of bacteria to algae, and consequently the efficiency of carbon, N and P removal in wastewater.^54^

**Table 3:** Main Characteristics of MPBR Suspensions

		mean ± SD	
parameter	units	R1	R2
pH	mg/L	7.8 ± 0.3	7.9 ± 0.3
Turbidity	NTU	302.9 ± 122.5	539.6 ± 111.3
CE	μS/cm	1430.6 ± 97.3	1493.9 ± 112.1
TSS	mg/L	648.8 ± 143.1	712.9 ± 76.0
VSS	mg/L	450.0 ± 125.9	557.9 ± 89.4
VSS/TSS	%	69.4 ± 1.0	78.3 ± 0.9
COD	mg/L	821.2 ± 241.4	989.7 ± 172.0
COD_s_	mg/L	77.7 ± 19.8	70.9 ± 32.4
DOC	mg/L	26.5 ± 4.1	23.9 ± 6.1
BPC	mg/L	12.2 ± 3.2	8.4 ± 3.9
BP	mg of VSS/L·d	47.6 ± 9.2	62.8 ± 3.9
COD_s_/VSS	mg of COD/mg of VSS	0.17 ± 0.05	0.12 ± 0.04
*n*	–	11	12
main biomass components (percentage composition)			
carbohydrates	%	63.1	55.4
proteins	%	15.7	21.9
lipids	%	16.0	11.5

**Figure 3:**
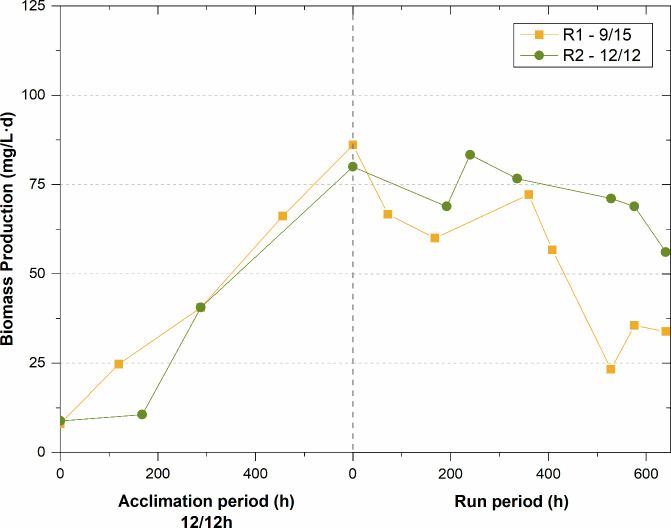
Biomass productivity evolution in both suspensions during the experimental runs and their corresponding acclimation periods.

Likewise, similar average concentrations of TSS were obtained, with values of 648.8 ± 143.0 and 712.9 ± 76.0 mg/L for R1 and R2, respectively. This is consistent with the total COD values, which were significantly higher in R2 than in R1 (989.7 ± 172.0 and 821.2 ± 241.4 mg/L, respectively). Regarding the nature of these solids, the VSS/TSS ratio was 69.4 ± 1.0% for R1 and 78.3 ± 0.9% for R2, indicating their predominantly organic character, but with the presence of inorganic components due to the nature of the feedwater, an effluent from a UASB reactor without post-treatment and with a high presence of suspended solids. Furthermore, the average particle size (*d*_50_) was 55.5 μm in R1 and 86.0 μm in R2, which may be attributed to an aggregation of microalgae and bacteria, favored by a greater presence of photosynthetic organisms due to the increased photoperiod. Yang et al.^55^ indicated that higher concentrations of dissolved oxygen (DO) due to the action of microalgae can enhance the activity of heterotrophic microorganisms, promoting the degradation of polysaccharides and increasing the protein/polysaccharide ratio. Several authors have observed that an increase in this ratio can lead to a reduction in electrostatic repulsion between cells and greater adhesiveness between autotrophic and heterotrophic microorganisms, stimulating bioflocs formation.^56–58^ These findings are consistent with the observed particle size and the percentage composition of the harvested biomass in R1 and R2.

#### 3.1.2 Characterization of the Phototrophic Organisms in the Suspension

Figure 4 shows the concentration of the main phototrophic organisms present in the suspensions during the two experimental runs: green algae, diatoms, and cyanobacteria. In general, the concentrations of green algae and diatoms increased during the trials in both photoperiods, while the number of cyanobacteria decreased slightly.

**Figure 4:**
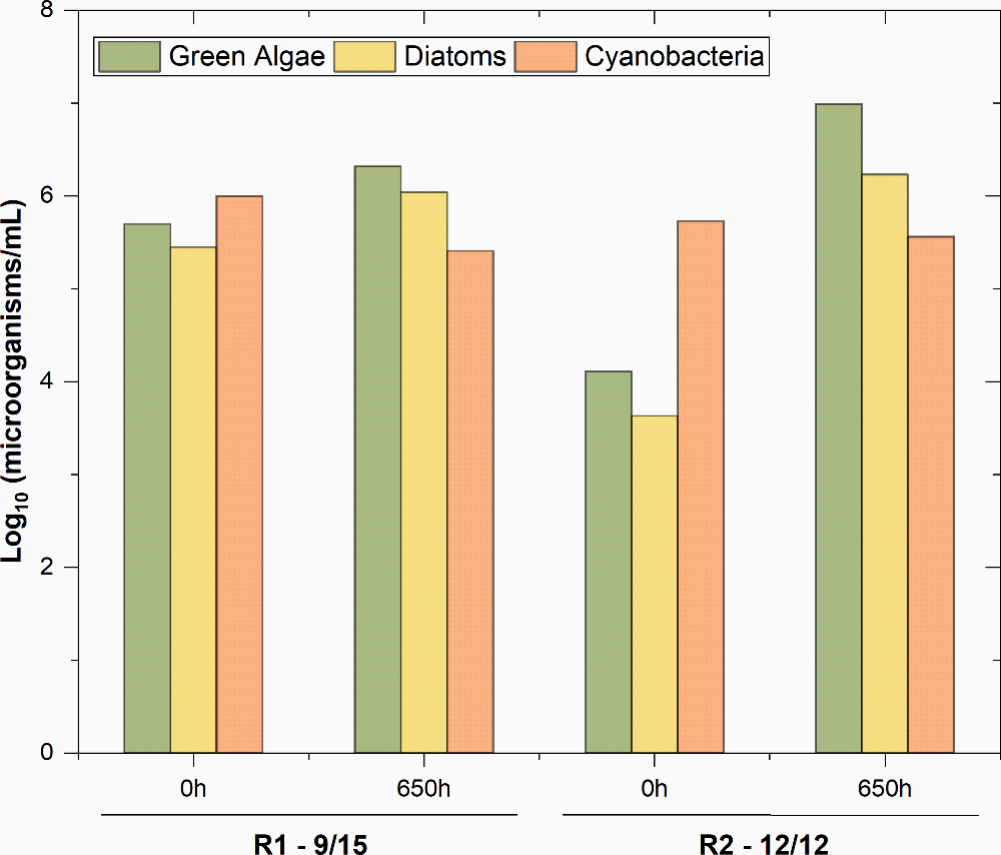
Counts of the main phototrophic microorganisms identified in both suspensions during the experimental runs.

Considering the relative abundance of the different species, for R2, the presence of green algae in the suspension increased during the study from 77.4% to 82.6%, mainly to the detriment of cyanobacteria, which decreased from 9.0% to only 3.0% of the species, while diatoms showed a stable temporal evolution (13.6% to 14.3%). In the case of the suspension of test R1, green algae decreased slightly from 64.0% to 60.7%, as did cyanobacteria, which decreased almost by half (from 15% to 7.5%). As for diatoms, they experienced an increase during the R1 experiment, increasing their relative presence by 10 points, from 21.1% to 31.8%.

These results are in line with the experimental study by Lürling et al.,^59^ which reported that the optimal temperatures for different species of chlorophytes and cyanobacteria were similar, ranging from 25 to 35 °C, exhibiting comparable growth rates. However, when the temperature was reduced to 20 °C, the authors observed that the overall growth rates of cyanobacteria were significantly lower compared to those of chlorophytes. This could explain the results observed in our experimental units, where the temperature was maintained at 18.5 ± 1.2 °C in both trials.

Regarding the differences observed between the runs, greater growth of green algae and diatoms was recorded in the 12/12 photoperiod compared to the 9/15 photoperiod, due to higher photosynthetic activity. Previous studies by Jia and Yuan^60,61^ confirmed that a longer light period resulted in a higher biomass growth rate, which is consistent with the physicochemical characterization of the suspension.

As for the different phototrophic species, organisms belonging to the genera *Navicula* (diatoms) and *Chlorella* (green algae) were identified as predominant as well as cyanobacteria. It is important to note that no inoculum was added to the system, as the aim was to work with the native microbial consortia present in the UASB effluent, thus reproducing more realistic and heterogeneous community dynamics. This is consistent with Nagarajan et al.,^19^ who emphasize that natural consortia evolve through processes of competition and cooperation, which inevitably lead to heterogeneous cultures under real wastewater conditions. Additionally, other organisms originating from the feedwater, such as nematodes, rotifers, annelids, and amoebas, were detected. The presence of other less prominent green algae genera, such as *Scenedesmus* and *Chaetophora*, was also noted, although *Chlorella* was the predominant genus in both experimental runs. *Scenedesmus* and *Chlorella* are genera commonly found in PBRs and MPBRs used for the treatment of anaerobic effluents both in laboratory and outdoor conditions, where the development of mixed cultures favors biomass production and reactor stability^29,45^

#### 3.1.3 Behavior of the Microalgae–Bacteria Consortium

Figure 5 shows the evolution of dissolved oxygen over time during the respirometric tests conducted for both biological suspensions at the end of each trial following the method by Rossi et al.^53^ During the first light phase (S-I: 0–30 min), which included the addition of ammonium and nitrite, a sharp increase in dissolved oxygen was observed due to intense microalgal photosynthetic activity. Simultaneously, oxygen consumption by ammonium-oxidizing (AOB) and nitrite-oxidizing bacteria (NOB) also occurred, resulting in slightly attenuated net oxygen production, as both photosynthesis and nitrification take place concurrently during this phase. This was further reflected during the dark phase (S-II: 30–40 min), when the dissolved oxygen decreased sharply due to the respiration of the microalgae, heterotrophic organisms, and nitrifying bacteria (AOB and NOB).

**Figure 5:**
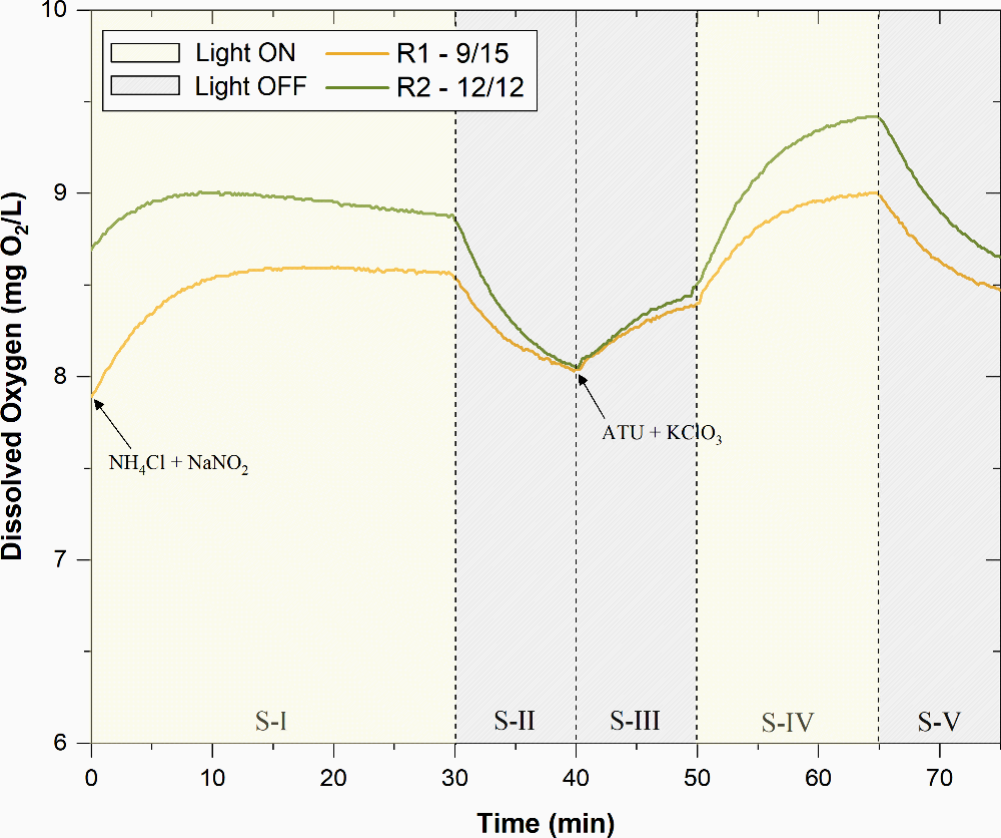
Results of respirometric tests for both biological suspensions (light on, yellow phase; light off, gray phase).

Indeed, the addition of nitrifying bacteria inhibitors (ATU and KClO_3_) during S-III (40–50 min) led to a partial recovery of the dissolved oxygen concentration in both biological suspensions, not due to oxygen production but rather to the reduced oxygen uptake combined with continuous aeration during the tests, reflecting only the respiration of microalgae and heterotrophic organisms. This would explain why both tests showed the same trend, ending S-III with values close to 8.5 mg/L. Then, in the final light phase (S-IV: 50–65 min), a sharp increase in dissolved oxygen was observed, reaching values of 9.0 and 9.4 mg/L in R1 and R2, respectively. Clearly, the absence of AOB and NOB resulted in higher net oxygen accumulation compared to phase I, where the microalgae compete for resources. This highlights the photosynthetic contribution of microalgae and the residual respiratory activity of heterotrophic organisms.

Additionally, the results confirm a greater presence of phototrophic organisms in the suspension when the photoperiod was increased from 9/15 (light on/off) to 12/12 (light on/off). Finally, in the last dark phase (S-V: 65–75 min), the DO concentrations in the suspension decreased asymptotically, mainly due to the respiration of microalgae and heterotrophs, since nitrification remained inhibited, reaching values similar to those reported at the end of S-III, and in a manner similar to the results obtained by Rossi et al.^53^ Following the methodology described in previous studies,^53,62^ specific oxygen production rate (sOPR) for microalgal photosynthetic (MAP) was higher for the suspension developed at 12/12 than at 9/15 (8.2 versus 5.0 mg of O_2_/gTSS·h). In addition, the role of nitrifying bacteria in the final suspensions was significant at 12/12 with a reported value of oxygen uptake rate per solids (sOUR) of 6.3 mg of O_2_/gTSS·h against 2.3 mg of O_2_/gTSS·h at 9/15. These results indicate the coexistence of microalgae and nitrifying bacteria in the suspensions, but with different population distributions. Some authors highlight the benefits of this consortium’s coexistence compared to traditional activated sludge systems, where photosynthetic oxygenation promotes the bacterial nitrification of ammonia present in wastewater, while organic matter is removed and the CO_2_ resulting from its decomposition is fixed, all while utilizing natural light as the main energy source.^63^ However, in order to achieve this goal, it is crucial to identify the operating conditions that favor a symbiotic rather than competitive interaction among microorganisms.^64^

### 3.2 Membrane Filtration Experiments

Figure 6a and Figure 6b show the evolution of the initial TMP (TMP_i_) and final TMP (TMP_f_) values during successive filtration cycles under R1 and R2 conditions, respectively. While the TMP_f_ represents the total fouling experienced by the membrane module before starting the backwashing cycles, TMP_i_ is usually related to internal residual fouling, which is typically caused by the adhesion of persistent organic compounds to the inner/outer surface of the membrane (i.e., gel layer, adsorption, or pore blocking). Therefore, cake layer cannot be detached and redispersed during physical cleanings. According to previous studies, the oscillations of the TMP curves in both runs (R1 and R2) are related to the light/dark cycles.^41^ In fact, the positive slope responds to the hours of light, while the hours of darkness are associated with negative slopes. This trend is conditioned by the microbiological activity of phototrophic organisms and the release of chemical compounds (such as polysaccharides, proteins, and fatty acids) that contribute to membrane fouling during the light periods.^34^

**Figure 6:**
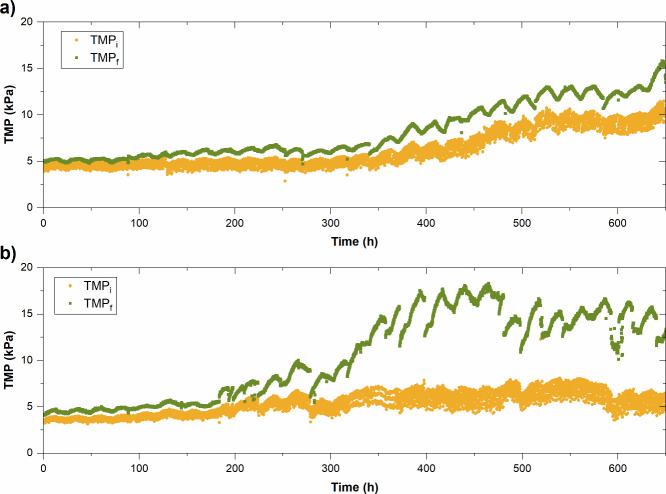
Evolution of TMP_i_ and TMP_f_ of (a) R1 with photoperiod 9/15 and (b) R2 with photoperiod 12/12.

In addition, the results in R1 show that TMP_i_ remains stable at 7.6 ± 0.6 kPa during the first 300 h (Figure 6a). Subsequently, a progressive increase in the internal residual fouling (0.03 kPa/h) was observed, coinciding with a sharp decrease in BP, from approximately 75 to 25 VSS mg/L·d. This change could be associated with stress or lysis of phototrophic biomass because of the decrease in light and the proliferation of nitrifying bacteria (section 3.4). In fact, previous studies indicate that one of the main limitations of anaerobic effluents is the proliferation of competing microorganisms, which can inhibit microalgae growth and even cause their death by limiting the exposure to light.^67,68^ Moreover, previous studies have noted that during competition for resources, microorganisms can experience stress and release extracellular polymeric substances (EPS) and soluble microbial products (SMP) into the bulk suspension. These compounds are the main contributors to residual and irreversible fouling, a phenomenon that can be exacerbated in the event of death and cellular lysis.^34,45,69,70^ The stress levels on the culture have been related to the COD_s_:VSS ratio,^62^ since it does not include changes in COD_s_ due to microalgae growth. In the current study, the COD_s_:VSS ratio was slightly higher for R1–9/15 than R2–12/12 confirming the development of heterotrophic bacteria and other superior microorganism growth in depletion of phototrophic ones. As expected, the TMP_f_ exhibited a parallel trend, stabilizing at values close to 17 kPa once the system reached pseudostationary operating conditions.

On the other hand, when the photoperiod was increased in R2 (Figure 6b), the predominant fouling of the membrane module was the reversible fraction (i.e., TMP_f_ – TMP_i_). Thus, while TMP_i_ remained relatively stable, with an average value of approximately 7.5 kPa, TMP_f_ increased rapidly to values above 20 kPa, and overall membrane fouling stabilized. This type of fouling is usually attributed to the formation of a reversible cake layer due to the deposition of larger particles. This is consistent with particle size distribution results, which exhibited 1.5 times higher *d*_50_ values in R2 than those in R1. This is also supported by the lower concentration of BPCs measured in R2 (8.4 ± 3.9 mg/L) compared to R1 (12.2 ± 3.2 mg/L). In addition, these results are consistent with previous studies where it has been observed that EPS and SMP can promote the aggregation of colloids and particles by acting as “binding agents”.^71^ The integration of these foulants into the floc structure, rather than remaining free in suspension, reduces the concentration of soluble matter in solution, as indicated by the COD_s_/COD ratio (0.095 and 0.072 for R1 and R2, respectively), thereby preventing residual internal fouling of the membrane module.

In fact, the bioflocculation process could be enhanced by the presence of a higher density of microalgae resulting from the increased exposure of the suspension to irradiance. From a filterability perspective, the results suggest that a balance is achieved between the microalgae and bacterial communities, when bioflocculation occurs naturally through the adhesion of microalgae to the surface of bacterial flocs, which improves process performance.^72^ Arcila and Buitrón^73^ reported that the formation of bioflocs between heterotrophic and autotrophic microorganisms is favored in the presence of nitrates and filamentous cyanobacteria, which facilitate the attachment of small colonies in the presence of EPS. These compounds are capable of creating a polymer network by forming bridges between bacteria, microalgae, and cyanobacteria.^72^ Therefore, the formation of bioflocs can reduce the release of soluble organic compounds in the mixed liquor, thus decreasing the level of internal fouling of the ultrafiltration membrane module.

Hence, the physical cleanings applied, backwashing aided with air sparging, were effective at preventing and controlling membrane fouling. Moreover, effective control of residual fouling allows the use of less aggressive chemical cleaning and reducing the frequency and/or working concentrations of chemical cleaning agents. Therefore, the membrane’s lifespan can be prolonged and operational costs reduced.

### 3.3 Characterization of Membrane Fouling after Long-Term Experiments

Figure 7 shows the contributions of different hydraulic resistances to the total membrane fouling during the long-term tests under R1 and R2. In both trials, the main contribution was related to loosen foulants, which can be detached from the membrane with physical cleaning methods, such as rinsing or backwashing. While the rinsing contribution was 40.8% in R1, its contribution increased to 65.3% when the photoperiod was extended to 12 h of light. Obviously, this physical cleaning only affects the external surface of the ZW-1 module, supporting the hypothesis of the build-up of a removable external cake layer in R2 as the primary fouling phenomena. On the other hand, the backwashing step suggests a greater presence of foulants inside the pores when the photoperiod was shorter (13.6% and 4.1% under R1 and R2, respectively). These results are consistent with a greater presence of colloidal and soluble matter in the bulk suspension, probably due to the stress and/or lysis of the phototrophic biomass. In fact, since the contribution of inorganic compounds to membrane fouling (i.e., foulants removed with citric acid) was practically negligible in both tests (<1%), a higher concentration of organic compounds, such as EPS and SMP, would explain the higher extent of residual fouling consolidation in R1 (37.6%) compared to R2 (23.7%). This is in accordance with the higher removal percentages observed for the attached organic matter in R2, when the membrane module was immersed in a 500 ppm solution of sodium hypochlorite (NaOCl). Furthermore, in both tests there was a fraction of persistent foulants that could not be eliminated at the end of the cleaning protocol, despite applying high concentrations of NaOCl (1000 ppm) for 24 h in the last step. Again, the degree of irreversible fouling was greater for R1 than R2 (17.8% and 13.1%, respectively).

**Figure 7:**
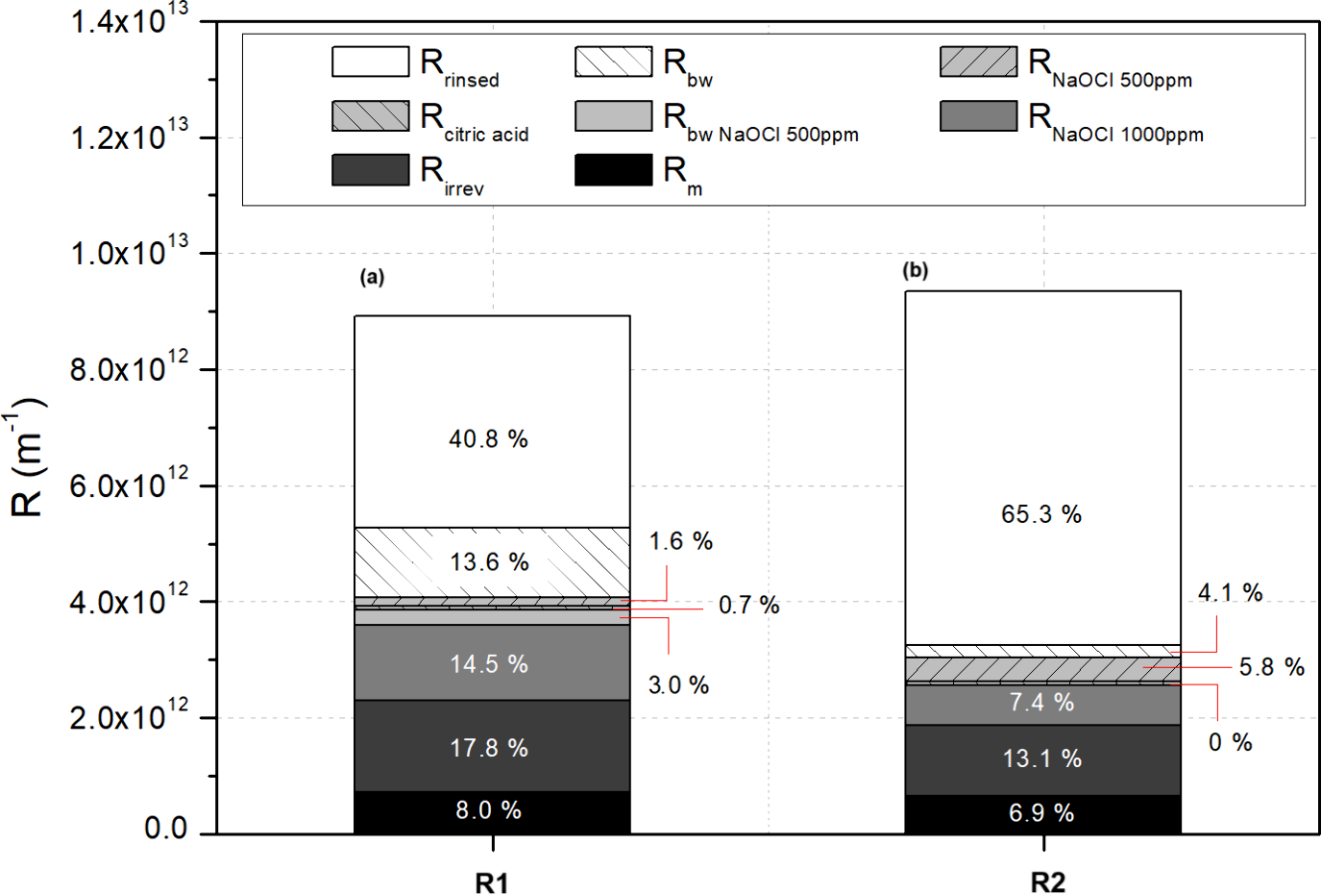
Hydraulic resistance contribution to membrane fouling in (a) R1 with photoperiod 9/15 and (b) R2 with photoperiod 12/12. *R*_rinsed_, rinsed membrane; *R*_bw_, following backwashing; *R*_NaOCl 500 ppm_, following 24 h immersion in 500 ppm of NaOCl; *R*_citric acid_, after 2 h in 6 g/L citric acid; *R*_bw NaOCl 500 ppm_, backwashed with 500 ppm of NaOCl; *R*_NaOCl 1000 ppm_, treated for 12 h in 1000 ppm of NaOCl; *R*_irrev_, irreversible fouling resistance; *R*_m_, intrinsic membrane resistance.

In summary, an increase in the photoperiod to 12/12 light/darkness led to a structural change in the fouling build-up, making it more reversible. An efficient control of reversible fouling using physical cleaning methods, which are less aggressive than the chemical ones, helps prolong the membrane’s lifespan while avoiding compromising its structural integrity.^74^ These results reinforce the significant role played by the external formation of a biocake. Indeed, previous studies indicate that the bioflocs formed by the presence of microalgae can have a significant impact on the filtration process, reducing the fouling potential of the membrane caused by the adsorption of small molecular-sized particles.^36,75^

Thus, the key role of the development of residual fouling during MPBR long-term operation should be noted, and future efforts must focus on optimizing the physical and chemical cleaning of this kind of system.

### 3.4 Removal of Pollutants and Quality of the Reclaimed Water

Figure 8 shows the evolution of the nitrification process during the filtration runs. The results indicate that 99% of dissolved ammonium was removed, and complete nitrification was observed after the acclimation phase in both experimental trials, regardless of the photoperiod applied. However, although ammonium was almost completely converted, the persistence of nitrate and nitrite in the effluent indicates that total nitrogen removal remained limited. As can be seen, the activity of the nitrifying bacteria was significant in both cases. However, the nitrification process evolved differently in each trial, with nitration being more stable in R2 (photoperiod with more daylight hours) than in R1. This difference could be attributed to higher photosynthetic activity of microalgae (more abundant in R2), as well as the dissolved oxygen concentration (greater than 8.5 mg/L) available for the development and activity of nitrifying bacteria. These results are consistent with the outcomes reported in the previous sections for R2: a higher concentration of green algae, diatoms, and cyanobacteria (microorganism counting, Figure 4) and increased photosynthetic activity (respirometric tests, Figure 5), confirming that the 12/12 photoperiod led to better nitrogen conversion and assimilation by phototrophic organisms. It should be noted that the high DO levels observed were largely a consequence of aeration for membrane scouring and mixing. However, the respirometric assays support that the contribution of photosynthetic oxygen was greater under the 12/12 h photoperiod, reinforcing the role of microalgae in supporting nitrification stability.

**Figure 8:**
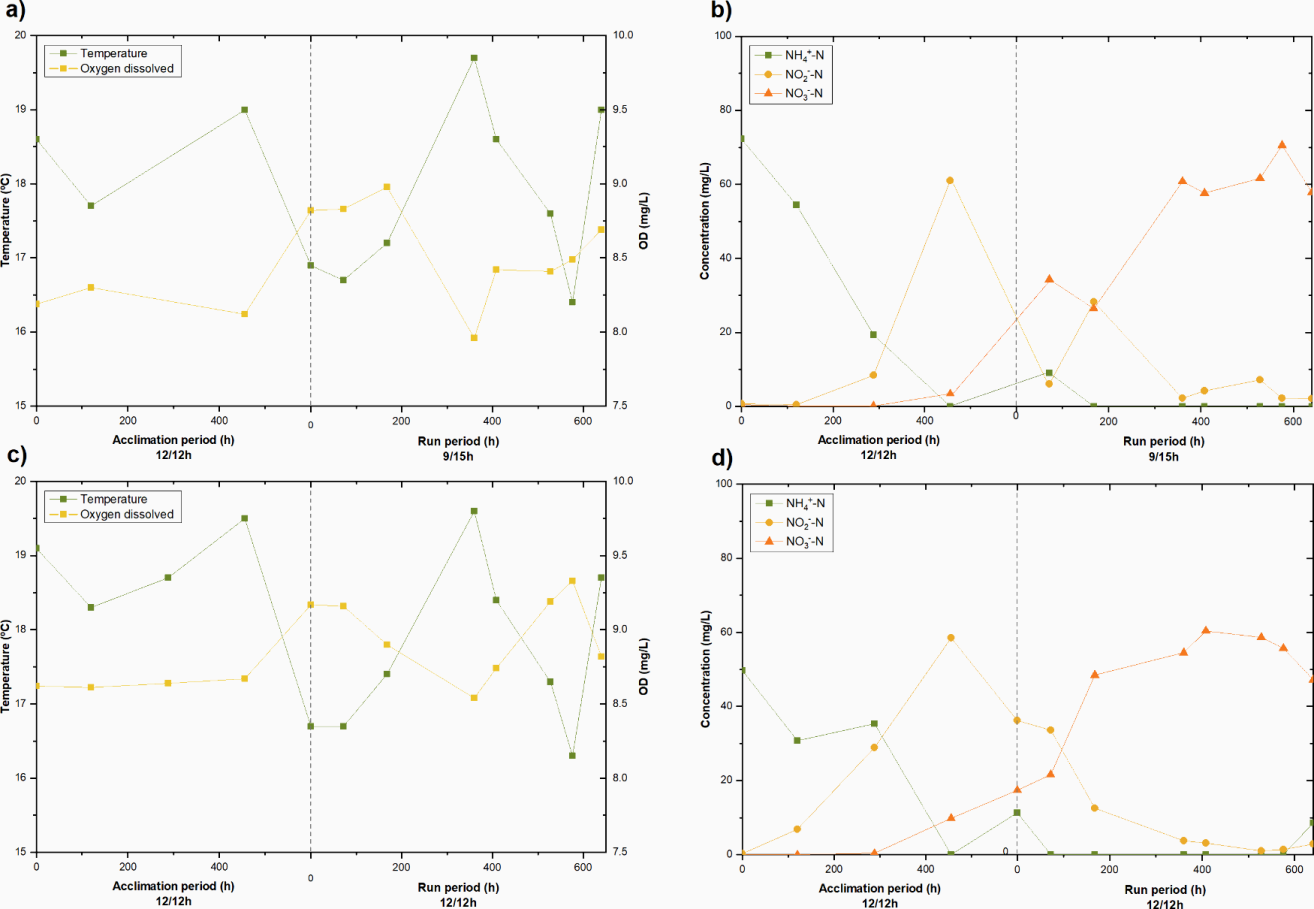
Evolution of dissolved oxygen and temperature for runs (a) R1 and (c) R2. Evolution of the nitrification process during experimental runs (b) R1 and (d) R2. Nitrogen compounds correspond to dissolved inorganic nitrogen species (DIN).

Table 4 shows the permeate quality in both MPBRs in terms of pH, turbidity, COD, DOC, and TP. Similar removal efficiencies for COD and DOC (73–76% and 64–67%, respectively) were reported in both tests, R1 and R2. Additionally, the membrane allowed the production of a solid-free permeate with final COD values in compliance with the European Urban Wastewater Directive (2024/3019/EEC).^4^ Regarding total phosphorus removal, it was 18.7% for R1 and 26.6% for R2, due to the greater proliferation of autotrophic microorganisms. Although these values are lower than those typically reported for the process, they are comparable to results obtained in studies that operated with low hydraulic and cellular retention times,^20^ which limits the complete assimilation of available nutrients in the feedwater. In our case, although the removal percentages were modest, they were similar to those reported by González-Camejo et al.,^44^ where phosphorus removal rates were 0.63 mg/L·d for the 9/15 regime and 0.95 mg/L·d for the 12/12 regime. These values are consistent with others reported in the literature, which typically range from 0.5 to 1.5 mg/L·d in algal–bacterial systems treating aerobic secondary effluents,^76,77^ and from 0.4 to 1.8 mg/L·d in MPBRs for reclaiming effluents from anaerobic membrane bioreactors (AnMBRs).^43–45^ Likewise, unlike studies that operated with anaerobic effluents free of particulate matter,^43^ the turbidity of the feedwater (52.9 ± 25.1 NTU) might have partially reduced light penetration, potentially affecting photosynthetic activity and phosphorus assimilation to some extent. This suggests that extending the solids retention time and optimizing light penetration could enhance overall phosphorus uptake efficiency.

**Table 4:** Main Characteristics of Permeate

		mean ± SD	
parameter	units	R1	R2
pH	–	8.0 ± 0.3	8.2 ± 0.3
turbidity	NTU	0.8 ± 0.4	0.7 ± 0.3
CE	μS/cm	1404.2 ± 95.3	1489.8 ± 99.5
COD	mg/L	44.4 ± 15.1	49.0 ± 19.7
DOC	mg/L	13.8 ± 0.9	14.6 ± 1.8
TP	mg/L	10.0 ± 1.5	9.1 ± 0.7

### 3.5 MPBR Performance without Gas Sparging under the 12/12 Light/Dark Photoperiod

In order to study the influence of the mixing system under a 12/12 light/dark photoperiod, a third experiment (R3) was carried out, maintaining the illumination conditions while replacing bottom aeration with continuous mechanical stirring (150 rpm). The results showed a biomass development similar to R2, with green algae and diatoms predominating over cyanobacteria (93–94% against 6–7%) (Figure 9a).

**Figure 9:**
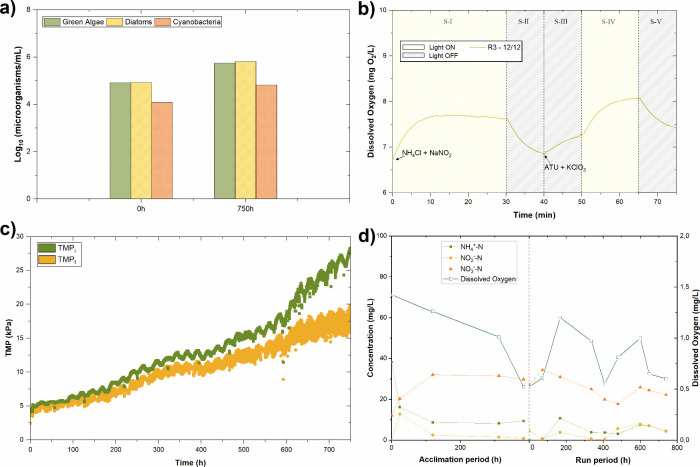
(a) Counts of the main phototrophic microorganisms identified in suspension during the experimental run R3 at different times. (b) Respirometric test for biological suspension R3 (light on, yellow phase; light off, gray phase). (c) Evolution of TMP_i_ and TMP_f_ of R3. (d) Evolution of nitrogen compounds corresponding to dissolved inorganic nitrogen species (DIN) during R3.

Regarding the respirometry assay, Figure 9b shows the evolution of DO over time for R3, where concentrations were slightly lower than those obtained in R2. This difference could be due to a lower presence of phototrophic microorganisms, which is consistent with the biomass concentration (Table 5) and the quantitative results (Figure 9a). This could be due to the feedwater fluctuations but also to the different hydrodynamic and mixing conditions without aeration. Nevertheless, both runs exhibited analogous trends, with very similar slopes in each of the different stages (S-I to S-V). In fact, sOPR and sOUR values of 7.8 mg of O_2_/ gTSS·h and 5.5 mg of O_2_/ gTSS·h were obtained for MAP and nitrifying bacteria, respectively, indicating the development of a microalgae and nitrifying bacteria population distribution equivalent to that observed in R2.

**Table 5:** Main Characteristics of MPBR Suspension and Permeate in Experimental Run R3

parameter	units	suspension	permeate
pH	mg/L	7.7 ± 0.1	7.9 ± 0.2
turbidity	NTU	237.5 ± 51.9	0.7 ± 0.4
CE	μS/cm	1333.3 ± 221.7	1316.8 ± 227.8
TSS	mg/L	438.4 ± 90.3	0.0
VSS	mg/L	351.8 ± 64.9	0.0
VSS/TSS	%	80.6 ± 0.5	–
COD	mg/L	683.1 ± 151.7	39.1 ± 15.0
COD_s_	mg/L	77.8 ± 26.9	39.1 ± 15.0
BPC	mg/L	38.6 ± 7.1	–
BP	mg of VSS/L·d	63.5 ± 1.9	–
COD_s_/VSS	mg of COD/mg of VSS	0.23 ± 0.07	–
*n*	–	8	8

Concerning membrane fouling, TMP values remained within the normal operating range for MBR systems, and considerably lower than the safety transmembrane pressure of the system (40 kPa). However, internal residual fouling was more pronounced (Figure 9c), with TMP_i_ values reaching approximately 15 kPa at the end of the assay. This increase could be attributed to a greater deposition of colloidal and soluble organic matter on the membrane, which is consistent with the higher BPC values recorded (Table 5). Several factors may contribute to this phenomenon, such as changes in hydrodynamic conditions caused by the absence of air sparging, which can alter flow patterns, turbulence dynamics and fouling propensity.^78^ Although mechanical stirring can achieve a more homogeneous mixing of the bulk suspension, some studies have shown that the biomass can be damaged by the mechanical stresses generated by agitation, but not by aeration.^79^ However, this effect depends on the type of microorganisms present in the mixed liquor, and on the operational scale, being more pronounced in bench units.^79^ In our study, the developed suspension during R2 exhibited a higher proportion of soluble COD (11%) of the total COD, which can be related to an increase of soluble and colloidal matter transported to the membrane surface.

Furthermore, a shift in the balance of the microalgae–bacteria consortium, resulting from a decreased DO concentration, could reduce the nitrifying activity of the bacteria and, consequently, the oxidation of readily biodegradable organic matter. Together, these phenomena could intensify internal residual fouling mechanisms such as gel layer formation or pore adsorption. This is consistent with the partial nitrification observed in Figure 9d, where the reduction in ammonium concentration reached 91.9 ± 6.8% under an average DO concentration of 1.02 mg of O_2_/L, maintained by the phototrophic microorganisms.

With regard to the nitrogen recovery, it should be noted that the excess air applied in experimental series R1 and R2 caused a total ammonium nitrification and did not allow for analysis of the role of phototrophic biomass in nitrogen recovery. In contrast, experimental test R3, carried out without aeration, showed that the phototrophic community developed under a 12/12 light/dark photoperiod, similar in distribution to that observed during experimental test R2, achieved a nitrogen removal rate (NRR) of 6.1 ± 1.3 mg/L·d. In this case, a combined process of nitrogen assimilation by biomass and partial ammonium nitrification allowed 28.4 ± 12.0% of the total nitrogen to be removed. These NRR values are similar to those obtained in experiments reported by González-Camejo,^45^ although in those cases, anaerobic effluents lacking turbidity from an anaerobic MBR were used, which favored light penetration and, in some cases, natural lighting was reinforced with artificial light. Regarding to the average phosphorus recovery ratio (PRR) achieved during experimental run R3, it was approximately 1.2 ± 0.5 mg/L·d, higher than those obtained in R1 and R2, and very close to values reported in various studies compiled by González-Camejo.^45^

In view of these results, MPBRs appear to have advantages over other types of reactors such as aerobic MBRs for advanced treatment of anaerobic secondary effluents. Thus, an aerobic MBR whose conventional configurations are designed primarily for the removal of high levels of organic matter (>90% COD), can remove ammonium by 10–30% through nitrification, as denitrification is limited to small endogenous microzones,^80,81^ and phosphorus removal, generally associated with biomass assimilation, is usually 10–20%, unless enhanced biological phosphorus removal (EBPR) or chemical dosing strategies are applied.^82,83^

To sum up, the results from experimental run R3 support the viability of the combination between UASB and MPBR at 12/12 light/dark photoperiod thanks to the phototrophic organisms that supply the oxygen needed for nitrogen recovery by assimilation and nitrification processes. In addition, this last experiment confirms the feasibility of employing alternative mixing modes of the biological suspension that avoid an air supply. This is an interesting aspect that opens new research opportunities, since bubble aeration, which is widely used, constitutes one of the highest energy costs in MBR systems due to its low efficiency and the limitations it presents for scale control, resulting from the irregular flow of bubbles generated by traditional aeration devices.^84^ In this sense, a recent study carried out for our team suggest that a UASB reactor fed with domestic wastewater and operating under optimal conditions could generate about 0.25 kWh·m^–3^ of electrical energy, which would supply approximately 50% of the energy demand of an MBR plant, typically around 0.4 kWh·m^–3^.^85^ Therefore, the UASB-MPBR combination shows great potential, and further pilot-scale studies must be carried out to optimize energy consumption and reduce the operational costs of the systems.

## 4 Conclusions

This study demonstrates that the performance of the MPBR treating UASB effluent is strongly governed by the interaction between the photoperiod and mixing strategy. Increasing illumination from 9/15 (R1) to 12/12 (R2 and R3) consistently enhanced microalgal activity, resulting in higher biomass productivity (from 47.6 ± 9.2 to 62.8 ± 3.9 mg and 63.5 ± 1.9 VSS/L·d, respectively), larger particles (*d*_50_ from 55 to 86 μm) and a higher organic fraction (VSS/TSS from ∼69% to ∼78–81%). This shift in biomass quality was accompanied by a clear restructuring of the phototrophic community: cyanobacteria remained more prevalent in R1 (7.5–15%), while extended illumination favored green algae and diatoms development, reaching 93–97% in R2 and R3, confirming the dominance of phototrophs under longer light exposure regardless of mixing mode.

These differences in biomass composition directly shaped fouling behavior. R1 accumulated more soluble and colloidal organic matter (COD_s_/VSS = 0.17 ± 0.05; BPC = 12.2 ± 3.2 mg/L), which led to the strongest internal residual fouling, reflected in the rise of TMP_i_ from 7.6 ± 0.6 kPa. In contrast, R2 exhibited lower soluble foulants (COD_s_/VSS = 0.12 ± 0.04; BPC = 8.4 ± 3.9 mg/L) and larger bioflocs, promoting a mainly reversible cake layer, with TMP_i_ remaining stable (∼7.5 kPa). When aeration was replaced by mechanical stirring (R3), fouling shifted again toward internal accumulation, with TMP_i_ reaching ∼15 kPa, showing that hydrodynamics, rather than photoperiod, determined the balance between reversible and residual foulants under 12/12 conditions.

Nutrient transformations further reflected these operational contrasts. Although both aerated runs, R1 and R2, achieved complete ammonium nitrification, nitrification stability improved under 12/12 (R2) due to higher photosynthetic oxygen supply (sOPR = 8.2; sOUR = 6.3 mg of O_2_/gTSS·h), whereas microalgal stress in R1 limited overall conversion. In configuration (R3) microalgae supplied enough oxygen to sustain partial nitrification (NH_4_^+^ removal 91.9 ± 6.8%), enabling a nitrogen recovery rate of 6.1 ± 1.3 mg N/L·d and the highest phosphorus recovery rate among the three runs (PRR = 1.2 ± 0.5 mg P/L·d).

Overall, extending illumination from 9/15 to 12/12 consistently improved biomass quality, nitrification stability, and fouling reversibility, while the mixing mode determined the extent of internal residual fouling under the same photoperiod. These findings confirm that R2 provides the most balanced operational window, whereas R3 demonstrates the feasibility of aeration-free MPBR operation, achieving acceptable biological performance at the expense of increased membrane fouling. The transfer of these results to larger scales will require consideration of light availability, mixing hydrodynamics, biomass retention, and energy demand. Pilot-scale studies are essential to validate the long-term performance and confirm the applicability of UASB-MPBR systems under real conditions.

## Data Availability

Data are available on request.

## References

[1] Cecconet D., Callegari A., Capodaglio A. G. (2022). UASB Performance and Perspectives
in Urban Wastewater Treatment at Sub-Mesophilic Operating Temperature. Water.

[2] Hejnic J., Dolejs P., Kouba V., Prudilova A., Widiayuningrum P., Bartacek J. (2016). Anaerobic Treatment
of Wastewater
in Colder Climates Using UASB Reactor and Anaerobic Membrane Bioreactor. Environ. Eng. Sci..

[3] Mainardis M., Buttazzoni M., Goi D. (2020). Up-flow anaerobic sludge blanket
(UASB) technology for energy recovery: A review on state-of-the-art
and recent technological advances. Bioengineering.

[4] Directive (EU) 2024/3019 of the European Parliament and of the Council of 27 November 2024 Concerning Urban Wastewater Treatment. European Commission, 2024. https://eur-lex.europa.eu/eli/dir/2024/3019/oj (accessed 2025-03-04).

[5] An Y., Yang F., Chua H. C., Wong F. S., Wu B. (2008). The integration
of methanogenesis with shortcut nitrification and denitrification
in a combined UASB with MBR. Bioresour. Technol..

[6] Buntner D., Sánchez A., Garrido J. M. (2013). Feasibility of combined UASB and
MBR system in dairy wastewater treatment at ambient temperatures. Chem. Eng. J..

[7] Sánchez A., Buntner D., Garrido J. M. (2013). Impact of methanogenic
pre-treatment
on the performance of an aerobic MBR system. Water Res..

[8] Zhang D., Lu P., Long T., Verstraete W. (2005). The integration of methanogensis
with simultaneous nitrification and denitrification in a membrane
bioreactor. Process Biochem..

[9] Lin H., Peng W., Zhang M., Chen J., Hong H., Zhang Y. (2013). A review on anaerobic
membrane bioreactors: Applications, membrane
fouling and future perspectives. Desalination.

[10] Mallick N. (2002). Biotechnological
potential of immobilized algae for wastewater N, P and metal removal:
A review. BioMetals.

[11] Rajagopal R., Choudhury M. R., Anwar N., Goyette B., Rahaman M. S. (2019). Influence
of pre-hydrolysis on sewage treatment in an Up-Flow Anaerobic Sludge
BLANKET (UASB) reactor: A review. Water.

[12] Regulation (EU) 2020/741 of the European Parliament and of the Council of 25 May 2020 on minimum requirements for water reuse. European Commission, 2020. https://eur-lex.europa.eu/eli/reg/2020/741/oj (accessed 2023-11-2023).

[13] Sustainable Use; Food and Agriculture Organisation, 2019. 10.1007/978-94-017-8801-4_211.

[14] Arbib Z., Ruiz J., Alvarez P., Garrido C., Barragan J., Perales J. A. (2012). *Chlorella stigmatophora* for Urban Wastewater Nutrient Removal and CO_2_ Abatement. Int. J. Phytorem..

[15] Beuckels A., Smolders E., Muylaert K. (2015). Nitrogen availability
influences
phosphorus removal in microalgae-based wastewater treatment. Water Res..

[17] Whitton R., Le Mével A., Pidou M., Ometto F., Villa R., Jefferson B. (2016). Influence of microalgal N and P composition
on wastewater
nutrient remediation. Water Res..

[18] Gonçalves A. L., Pires J. C. M., Simões M. (2017). A review on
the use of microalgal
consortia for wastewater treatment. Algal Res..

[19] Nagarajan D., Lee D. J., Varjani S., Lam S. S., Allakhverdiev S. I., Chang J. S. (2022). Microalgae-based
wastewater treatment – Microalgae-bacteria
consortia, multi-omics approaches and algal stress response. Sci. Total Environ..

[20] Luo Y., Le-Clech P., Henderson R. K. (2018). Assessment
of membrane photobioreactor
(MPBR) performance parameters and operating conditions. Water Res..

[21] Chen X., Li Z., He N., Zheng Y., Li H., Wang H., Wang Y., Lu Y., Li Q., Peng Y. (2018). Nitrogen and
phosphorus removal from anaerobically digested wastewater by microalgae
cultured in a novel membrane photobioreactor. Biotechnol. Biofuels.

[22] Marbelia L., Bilad M. R., Passaris I., Discart V., Vandamme D., Beuckels A., Muylaert K., Vankelecom I. F. J. (2014). Membrane
photobioreactors for integrated microalgae cultivation and nutrient
remediation of membrane bioreactors effluent. Bioresour. Technol..

[23] Praveen P., Loh K. C. (2016). Nitrogen and phosphorus
removal from tertiary wastewater
in an osmotic membrane photobioreactor. Bioresour.
Technol..

[24] Gao F., Peng Y. Y., Li C., Cui W., Yang Z. H., Zeng G. M. (2018). Coupled nutrient
removal from secondary effluent and
algal biomass production in membrane photobioreactor (MPBR): Effect
of HRT and long-term operation. Chem. Eng. J..

[25] Kumar A., Yuan X., Sahu A. K., Ergas S. J., Van Langenhove H., Dewulf J. (2010). A hollow fiber membrane
photo-bioreactor for CO_2_ sequestration from combustion
gas coupled with wastewater
treatment: A process engineering approach. J.
Chem. Technol. Biotechnol..

[26] Saini N., Dhull P., Pal M., Manzoor I., Rao R., Mushtaq B., Aamir M. (2024). Algal Membrane Bioreactors for Efficient
Removal of Emerging Contaminants and Resource Recovery: Current Advances
and Future Outlook. J. Environ. Chem. Eng..

[27] Najafi
Chaleshtori S., Shamskilani M., Babaei A., Behrang M. (2022). Municipal
wastewater treatment and fouling in microalgal-activated sludge membrane
bioreactor: Cultivation in raw and treated wastewater. J. Water Process Eng..

[28] Ruiz-Martinez A., Martin Garcia N., Romero I., Seco A., Ferrer J. (2012). Microalgae
cultivation in wastewater: Nutrient removal from anaerobic membrane
bioreactor effluent. Bioresour. Technol..

[29] Pachés M., Martínez-Guijarro R., González-Camejo J., Seco A., Barat R. (2020). Selecting
the most suitable microalgae
species to treat the effluent from an anaerobic membrane bioreactor. Environ. Technol..

[30] Ding M., Wang C., Woo Bae S., Yong Ng H. (2022). Enhanced nutrient
removal
and bioenergy production in microalgal photobioreactor following anaerobic
membrane bioreactor for decarbonized wastewater treatment. Bioresour. Technol..

[31] Gaio J., Lora N. L., Iltchenco J., Magrini F. E., Paesi S. (2023). Seasonal characterization
of the prokaryotic microbiota of full-scale anaerobic UASB reactors
treating domestic sewage in southern Brazil. Bioprocess Biosyst. Eng..

[32] Rattier M., Jimenez J. A., Miller M. W., Dhanasekar A., Willis J., Keller J., Batstone D. (2022). Long-term comparison
of pilot UASB and AnMBR systems treating domestic sewage at ambient
temperatures. J. Environ. Chem. Eng..

[33] Azizi S., Hashemi A., Pajoum Shariati F., Tayebati H., Keramati A., Bonakdarpour B., Shirazi M. M. A. (2021). Effect of different light-dark cycles
on the membrane fouling, EPS and SMP production in a novel reciprocal
membrane photobioreactor (RMPBR) by *C. vulgaris* species. J. Water Process Eng..

[34] Novoa A. F., Vrouwenvelder J. S., Fortunato L. (2021). Membrane Fouling in Algal Separation
Processes: A Review of Influencing Factors and Mechanisms. Front. Chem. Eng..

[35] Bilad M. R., Discart V., Vandamme D., Foubert I., Muylaert K., Vankelecom I. F. J. (2014). Coupled cultivation and pre-harvesting
of microalgae
in a membrane photobioreactor (MPBR). Bioresour.
Technol..

[36] Liao Y., Bokhary A., Maleki E., Liao B. (2018). A review of membrane
fouling and its control in algal-related membrane processes. Bioresour. Technol..

[37] Ferrera E., Ruigómez I., Vera L. (2022). Preliminary Study of Up-Flow Anaerobic
Sludge Blanket (UASB) Technology for Energy Recovery from Domestic
Wastewater. Renewable Energy Power Qual. J..

[38] Bandara W.M.K.R.T.W., Kindaichi T., Satoh H., Sasakawa M., Nakahara Y., Takahashi M., Okabe S. (2012). Anaerobic treatment of municipal
wastewater at ambient temperature: Analysis of archaeal community
structure and recovery of dissolved methane. Water Res..

[39] Essam T., ElRakaiby M., Hashem A. (2013). Photosynthetic based algal-bacterial
combined treatment of mixtures of organic pollutants and CO_2_ mitigation in a continuous photobioreactor. World J. Microbiol. Biotechnol..

[40] Jacob-Lopes E., Scoparo C. H. G., Lacerda L. M. C. F., Franco T. T. (2009). Effect of light
cycles (night/day) on CO_2_ fixation and biomass production
by microalgae in photobioreactors. Chem. Eng.
Process..

[41] González E., Díaz O., Ruigómez I., de Vera C. R., Rodríguez-Gómez L. E., Rodríguez-Sevilla J., Vera L. (2017). Photosynthetic bacteria-based
membrane bioreactor as post-treatment of an anaerobic membrane bioreactor
effluent. Bioresour. Technol..

[42] Ferrera E., Ruigómez I., Vela-Bastos C., Ferreira A., Gouveia L., Vera L. (2024). Resources
recovery from domestic wastewater by a combined process:
anaerobic digestion and membrane photobioreactor. Environ. Sci. Pollut. Res..

[43] Viruela A., Robles Á., Durán F., Ruano M. V., Barat R., Ferrer J., Seco A. (2018). Performance
of an outdoor membrane
photobioreactor for resource recovery from anaerobically treated sewage. J. Cleaner Prod..

[44] González-Camejo J., Barat R., Ruano M. V., Seco A., Ferrer J. (2018). Outdoor flat-panel
membrane photobioreactor to treat the effluent of an anaerobic membrane
bioreactor, Influence of operating, design, and environmental conditions. Water Sci. Technol..

[45] González-Camejo J., Jiménez-Benítez A., Ruano M. V., Robles A., Barat R., Ferrer J. (2019). Optimising an outdoor membrane photobioreactor
for tertiary sewage treatment. J. Environ. Manage..

[46] Honda R., Teraoka Y., Noguchi M., Yang S. (2017). Optimization of Hydraulic
Retention Time and Biomass Concentration in Microalgae Biomass Production
from Treated Sewage with a Membrane Photobioreactor. J. Water Environ. Technol..

[47] Standard Methods for the Examination of Water and Wastewater, 21st ed.; American Public Health Association/American Water Works Association/Water Environment Federation: Washington, DC, 2005.

[48] Ferreira A. F., Ferreira A., Dias A. P. S., Gouveia L. (2020). Pyrolysis of *Scenedesmus obliquus* Biomass Following the Treatment
of Different Wastewaters. Bioenergy Res..

[49] Ruigómez I., González E., Guerra S., Rodríguez-Gómez L. E., Vera L. (2017). Evaluation of a novel physical cleaning strategy based on HF membrane
rotation during the backwashing/relaxation phases for anaerobic submerged
MBR. J. Membr. Sci..

[50] Madoni P. (1994). A sludge biotic
index (SBI) for the evaluation of the biological performance of activated
sludge plants based on the microfauna analysis. Water Res..

[51] Rodríguez-González, E. ; Oria, L. I. ; Fernández Moriña, N. ; Rodríguez, M. D. S. ; Gamero, C. J. Guía Metodológica para la Elaboración de Análisis Microbiológicos de Fangos Activados de Estaciones de Depuradoras de Aguas Residuales; Grupo Bioindicación Sevilla, 2002.

[52] Berk, S. G. ; Gunderson, J. H. Wastewater Organisms: A Color Atlas; Routledge, 1993.

[53] Rossi S., Bellucci M., Marazzi F., Mezzanotte V., Ficara E. (2018). Activity assessment of microalgal-bacterial
consortia
based on respirometric tests. Water Sci. Technol..

[54] Mohsenpour S. F., Hennige S., Willoughby N., Adeloye A., Gutierrez T. (2021). Integrating
micro-algae into wastewater treatment: A review. Sci. Total Environ..

[55] Yang B., Yan Y., Jia Y., Chen B., Khanal S. K., Shu W.-s., Lu H. (2025). Optimizing
formation of microalgal-bacterial granular sludge for
aquaculture wastewater treatment. Chem. Eng.
J..

[56] Guo G., Li Y., Zhou S., Chen Y., Urasaki K., Qin Y., Kubota K., Li Y.-Y. (2022). Long term operation performance and
membrane fouling mechanisms of anaerobic membrane bioreactor treating
waste activated sludge at high solid concentration and high flux. Sci. Total Environ..

[57] Zhou Y., Cui X., Wu B., Wang Z., Liu Y., Ren T., Xia S., Rittmann B. E. (2024). Microalgal extracellular polymeric substances (EPS)
and their roles in cultivation, biomass harvesting, and bioproducts
extraction. Bioresour. Technol..

[58] Xiong Y., Liu Y. (2013). Importance of extracellular
proteins in maintaining structural integrity
of aerobic granules. Colloids Surf., B.

[59] Lürling M., Eshetu F., Faassen E. J., Kosten S., Huszar V. L. M. (2013). Comparison
of cyanobacterial and green algal growth rates at different temperatures. Freshwater Biol..

[60] Jia H., Yuan Q. (2016). Removal of nitrogen
from wastewater using microalgae and microalgae–bacteria
consortia. Cogent Environ. Sci..

[61] Jia H., Yuan Q. (2018). Ammonium removal using
algae–bacteria consortia: the effect
of ammonium concentration, algae biomass, and light. Biodegradation.

[62] González-Camejo J., Aparicio S., Jiménez-Benítez A., Pachés M., Ruano M. V., Borrás L., Barat R., Seco A. (2020). Improving membrane photobioreactor
performance by reducing light path: operating conditions and key performance
indicators. Water Res..

[63] Foladori P., Petrini S., Andreottola G. (2020). Heliyon How
suspended solids concentration
affects nitri fi cation rate in microalgal-bacterial photobioreactors
without external aeration. Heliyon.

[64] Aditya L., Mahlia T. M. I., Nguyen L. N., Vu H. P., Nghiem L. D. (2022). Microalgae-bacteria
consortium for wastewater treatment and biomass production. Sci. Total Environ..

[67] Chen X., Li Z., He N., Zheng Y., Li H., Wang H., Wang Y., Lu Y., Li Q., Peng Y. (2018). Nitrogen and
phosphorus removal from anaerobically digested wastewater by microalgae
cultured in a novel membrane photobioreactor. Biotechnol. Biofuels.

[68] Gao F., Yang Z.-H., Li C., Wang Y.-j., Jin W.-h., Deng Y.-b. (2014). Concentrated microalgae cultivation in treated sewage
by membrane photobioreactor operated in batch flow mode. Bioresour. Technol..

[69] Lin H., Zhang M., Wang F., Meng F., Liao B. Q., Hong H., Chen J., Gao W. (2014). A critical review of
extracellular polymeric substances (EPSs) in membrane bioreactors:
Characteristics, roles in membrane fouling and control strategies. J. Membr. Sci..

[70] Garza-Rodríguez Z. B., Hernández-Pérez J., Santacruz A., Jacobo-Velázquez D. A., Benavides J. (2022). Prospective
on the application of abiotic stresses to enhance the industrial production
of exopolysaccharides from microalgae. Curr.
Res. Biotechnol.

[71] Ferrera E., Ruigómez I., Vera L. (2024). Pilot scale application
of a rotating
hollow fibre membrane for direct membrane filtration of domestic wastewater. J. Water Process Eng..

[72] Satiro J., dos Santos Neto A. G., Marinho T., Sales M., Marinho I., Kato M.T., Simões R., Albuquerque A., Florencio L. (2024). The Role of the Microalgae–Bacteria
Consortium
in Biomass Formation and Its Application in Wastewater Treatment Systems:
A Comprehensive Review. Appl. Sci..

[73] Arcila J. S., Buitrón G. (2017). Influence
of solar irradiance levels on the formation
of microalgae-bacteria aggregates for municipal wastewater treatment. Algal Res..

[74] Aslam M., Charfi A., Lesage G., Heran M., Kim J. (2017). Membrane bioreactors
for wastewater treatment: A review of mechanical cleaning by scouring
agents to control membrane fouling. Chem. Eng.
J..

[75] Low S. L., Ong S. L., Ng H. Y. (2016). Characterization of membrane fouling
in submerged ceramic membrane photobioreactors fed with effluent from
membrane bioreactors. Chem. Eng. J..

[76] Arias D. M., Uggetti E., García-Galán M. J., García J. (2017). Cultivation
and selection of cyanobacteria in a closed photobioreactor used for
secondary effluent and digestate treatment. Sci. Total Environ..

[77] Posadas E., García-Encina P. A., Soltau A., Domínguez A., Díaz I., Muñoz R. (2013). Carbon and nutrient removal from
centrates and domestic wastewater using algal-bacterial biofilm bioreactors. Bioresour. Technol..

[78] Meng F., Chae S. R., Drews A., Kraume M., Shin H. S., Yang F. (2009). Recent advances in
membrane bioreactors (MBRs): Membrane fouling
and membrane material. Water Res..

[79] Nienow A. W. (2009). Scale-Up
Considerations Based on Studies at the Bench Scale in Stirred Bioreactors. J. Chem. Eng. Jpn..

[80] Judd, S. ; Judd, C. The MBR Book: Principles and Applications of Membrane Bioreactors for Water and Wastewater Treatment; Elsevier/Butterworth-Heinemann, 2011.

[81] Trussell R.
S., Merlo R. P., Hermanowicz S. W., Jenkins D. (2006). The effect of organic
loading on process performance and membrane fouling in a submerged
membrane bioreactor treating municipal wastewater. Water Res..

[82] Meng F., Drews A., Mehrez R., Iversen V., Ernst M., Yang F., Jekel M., Kraume M. (2009). Occurrence,
source,
and fate of dissolved organic matter (DOM) in a pilot-scale membrane
bioreactor. Environ. Sci. Technol..

[83] Al-Asheh S., Bagheri M., Aidan A. (2021). Membrane bioreactor
for wastewater
treatment: A review. Case Stud. Chem. Environ.
Eng..

[84] Mayer M., Braun R., Fuchs W. (2006). Comparison
of various aeration devices
for air sparging in crossflow membrane filtration. J. Membr. Sci..

[85] Krzeminski P., Leverette L., Malamis S., Katsou E. (2017). Membrane bioreactors
– A review on recent developments in energy reduction, fouling
control, novel configurations, LCA and market prospects. J. Membr. Sci..

